# Knowledge, attitudes, and experiences in suicide assessment and management: a qualitative study among primary health care workers in southwestern Uganda

**DOI:** 10.1186/s12888-022-04244-z

**Published:** 2022-09-12

**Authors:** Godfrey Z. Rukundo, Edith K. Wakida, Samuel Maling, Mark M. Kaggwa, Baker M. Sserumaga, Letizia M. Atim, Clara D. Atuhaire, Celestino Obua

**Affiliations:** 1 Department of Psychiatry, Mbarara University of Science and Technology, Mbarara, Uganda; 2 African Center for Suicide Prevention and Research, Mbarara, Uganda; 3 Office of Research Administration, Mbarara University of Science and Technology, Mbarara, Uganda; 4 Department of Psychiatry and Behavioral Neurosciences, McMaster University, Hamilton, Canada; 5 Department of Pharmacology and Therapeutics, Mbarara University of Science and Technology, Mbarara, Uganda

**Keywords:** Suicide assessment, management of suicidal behaviors, primary health care, Uganda

## Abstract

**Background:**

Suicide is one of the leading causes of death globally, with over 75% of all suicides occurring in low-and middle-income Countries. Although 25% of people have contact with their health care workers before suicide attempts, most never receive proper suicide assessment and management. We explored primary care health workers' knowledge, attitudes, and experiences in evaluating and managing suicidality in structured primary healthcare services in Uganda.

**Methods:**

This was a cross-sectional qualitative study among health workers in southwestern Uganda from purposively selected health facilities. A semi-structured interview guide was used, and data were analyzed using thematic analysis.

**Results:**

The in-depth interviews were conducted with 18 individuals (i.e., five medical doctors, two clinical officers, two midwives, and nine nurses) from 12 health facilities in the five selected districts. Four themes emerged from the discussions: a) Knowledge and attitudes of primary healthcare workers in the assessment and management of suicidality, b) Experiences in the assessment and management of suicidality, c) challenges faced by primary healthcare workers while assessing and managing suicidality, and d) Recommendations for improving assessment and management of suicidality in PHC.

Most participants were knowledgeable about suicide and the associated risk factors but reported challenges in assessing and managing individuals with suicide risk. The participants freely shared individual experiences and attitudes in the assessment and management of suicide. They also proposed possible ways to improve the evaluation and management of suicidality in PHC, such as setting up a system of managing suicidality, regularizing community sensitization, and training health workers.

**Conclusion:**

Suicidality is commonly encountered by primary health care workers in Uganda who struggle with its assessment and management. Improving the knowledge and attitudes of primary health care workers would be a big step towards ensuring equitable services.

## Introduction

Suicide is one of the leading global causes of death, with over 75% of all suicides occurring in Low-and Middle-Income Countries (LMICs) [1, 2]. For each suicide, there are many more persons with serious suicidal ideation and nonfatal attempts [3, 4]. Of the individuals who died by suicide or attempted in Australia, approximately 25% contacted health care providers in the previous month [5]. However, no such data exists for individuals in sub-Sahara Africa (SSA), such as Uganda. However, most individuals with suicidal behaviors in SSA do not receive appropriate assessment and management for their suicide risk [6, 7]. This may be attributed to health providers’ knowledge gaps and negative attitudes towards suicide [7, 8]. Yet, primary health care workers (PHCWs) are the gatekeepers for health care who are expected to screen for all diseases and either offer appropriate care or referral [9, 10]. Admitting individuals with severe suicidal ideation, plan, or attempt is common practice. The admission usually prevents eventual suicide in the short term and for diagnosis and management of the underlying psychiatric or medical conditions.

Many health workers often do not ask about suicidality in their routine patient assessments for fear that asking someone if they were thinking about suicide would “put the idea in their head,” or it is considered rude [4, 11]. Besides, suicide is not part of the routine checkup in primary health care (PHC) settings. The PHCWs find it hard to ask about suicidality due to the stigma surrounding suicidality [12]. Some attitudes include considering suicide an immoral or criminal act, thereby blaming the individuals presenting with suicidality [13]. Such attitudes are reinforced by the fact that suicide is illegal in Uganda, and individuals who attempt suicide are jailed for a minimum of six months [8, 14]. In addition, culturally, individuals who die by suicide are also punished (the dead body is beaten) and not given a proper burial to teach community members not to get involved in similar behaviors [15, 16].

Previous suicide research in Uganda and other sub-Saharan countries has not focused on health professionals', knowledge, skills and practices but have easily focused on suicide prevalence and associated factors [14, 15, 17–20]. In this paper, therefore, we explored the knowledge, attitudes, and experiences of PCHWs in assessing and managing suicidality in structured PHC services in southwestern Uganda.

## Methods

### Study design

This cross-sectional qualitative study examined the knowledge and experiences of PHCWs in assessing and managing suicidality in structured primary healthcare services [21]. We used a semi-structured interview guide with open-ended questions that permitted respondents to share experiences.

### Study setting and context

This study was conducted in five districts in Southwestern Uganda (i.e., Mbarara, Sheema, Isingiro, Rwampara, and Ntungamo). Provision of mental health services in Uganda begins at a sub-county level health center (HC III), after which referrals can be made to county level HC IV, district hospitals, regional referral hospitals, and finally to the national referral mental hospital [22]. Each HC III or higher is expected to have a mental health worker with the capacity to assess and manage various mental health conditions [23–25]. We purposively selected two general hospitals (Itojo hospital in Ntungamo district and Kitagata hospital in Sheema district) and 12 health centers (Health center III and IV) based on having high patient turnover from a rural settings with guidance from the District Health Officers (DHOs) in each district. The study health centers in Mbarara district were Bwizibwera HC IV and Rubaya HC III; Sheema district: Shuuku HC IV and Kabwohe HC IV; Rwampara district: Kinoni HC IV and Ndeija HC III; Ntungamo district: Rubaare HC IV; Isingiro district: Nyamuyanja HC IV, Kabuyanda HCIV and Rwekubo HCIV.

### Study participants

The study participants with whom we reached content saturation were purposively selected based on (i) their cadre, (ii) level of experience, (iii) most senior staff involved in the day-to-day patient assessment and care, and (iv) willingness to participate in the study. We excluded health care workers with professional training in mental health (such as psychiatric nurses or psychiatric clinical officers) – these are rarely involved in screening patients who attend the PHC and usually receive referrals from their peers. In addition, they have relatively higher levels of knowledge than other health professionals due to their training, which may not reflect the experiences of the vast number of health workers. The In-Charges identified these participants at each health facility, and the in-charge was first contacted before starting data collection. The cadres of the participants included medical doctors (*n* = 5), clinical officers (*n* = 2), midwives (*n* = 2), and nine general nurses (Table 1). We conducted face-to-face interviews with the most senior person available on the day of the interview.

**Table 1:** Demographics characteristics of participants in the study

Participant ID	Gender	Level of Education ^**a**^	Level of HF ^**b**^	Health Cadre ^**c**^
AL001	F	MBChB	HC IV	MO
AL002	M	MBChB	HCIV	MO
AL003	F	Diploma CM	HC IV	CO
AL004	M	MBChB	HCIV	MO
AL005	M	BNS	HC III	NO
AL006	M	BNS	Hospital	NO
AL007	F	Nursing Certificate	HC III	Enrolled Midwife
AL008	F	Nursing Certificate	HC III	Enrolled Nurse
AL009	M	Diploma DCM	HC IV	CO
AL010	F	BNS	HC IV	NO
AL011	M	BNS	Hospital	NO
AL012	F	Certificate Nursing	HCIII	Enrolled Nurse
AL013	F	BNS	HCIV	NO
AL014	M	MBChB	HCIV	MO
AL015	F	Nursing Diploma	HC III	Assistant NO
AL016	F	Certificate Midwifery	HC IV	Enrolled Midwife
AL017	F	Diploma Nursing	Hospital	Assistant NO
AL018	M	MBChB	Hospital	MS

^a^ *MBChB* medical degree, *BNS* nursing degree, *DCM* diploma clinical medicine

^b^ *HF* Health Facility

^c^ *CO* Clinical Officer, *MO* Medical Officer, *MS* Medical Superintendent, *NO* Nursing Officer

### Data collection

The research team developed a semi-structured in-depth interview guide based on the review of the literature [7, 26] and expert opinions from psychiatrists at the Mbarara University of science and technology to collect data from the PHCWs. The questions were: Knowledge and Attitudes: (i*) What are the common causes of suicidality?* (ii) *What would you do if you developed features of suicidality?* (iii) *What should be done to help people with suicidality presenting to primary health care centers?* (iv) *How best can suicidality be managed or treated?* (v) *What else would you like to say about suicidality in primary health care settings?* Experiences: (i) *How has your experience been working with people presenting with suicidality?* (ii) *What has been the most challenging experience in the assessment and management of suicidality?*

We asked the participants to freely share any other information they wanted to say about the discussion points. We also asked them to suggest recommendations to improve suicide assessment and management in primary health care settings. Here are the questions: *How has your experience been working with people presenting with suicidality? What else would you like to say about suicidality in primary health care settings?*

The interviews were conducted in English by research assistants (psychiatry residents – MMK, BMS, and LMA) trained in qualitative data collection who audio recorded, wrote the field notes and memos. All interviews were conducted in person at the respective health facilities after the PHCWs were done with their day-to-day activities of managing patients. Each interview lasted an average of 20–30 minutes. All methods were performed following the relevant guidelines and regulations (i.e., national, international, institutional, and the Helsinki Declaration).

### Data management and quality assurance

The research assistants transcribed all audio recordings, and GZR checked the transcripts against the audio recordings to confirm the correctness and ensure no data loss. GZR securely stored the recordings, transcription, field notes, and memos in a lockable office that was only accessible to the research team. In-field discussions were held daily to discuss emerging ideas/themes and capture representative views from the different cadres of health workers, districts, and levels of health care facilities.

### The rigor of this study

Guba’s model of trustworthiness was employed [27]. This model includes credibility, transferability, conformability, and dependability. Credibility was ensured by establishing rapport with participants and telling their stories and unique experiences throughout the study. Conformability was done by evaluating interview questions to ensure they were open-ended and not leading. Codes used for each respondent were allowed to match each respondent’s description properly. A clear trail was created where all steps taken in the research were outlined and made available to members of the research team for scrutiny. Dependability was achieved by accurately documenting the processes undertaken. This detailed documentation will enable the readers to ascertain that appropriate research methods were adhered to and provide future researchers with the depth of information needed for replication in other settings [28]. Transferability was achieved by eliciting detailed descriptions of study methods, adequate sampling, and the purposive selection of the study participants [27].

### Data analysis

All transcriptions were anonymized before analysis. GZR and CDA conducted a thematic content analysis [29, 30] in consultation with EKW (expert in qualitative research) and CO (senior researchers). Transcripts were independently coded by GZR and CDA using codes generated from the data, and disagreements in the coding were resolved at each step. The process involved the identification of similarities and differences through the participants’ narratives. Coding was done aided by Atlas.ti version 8, grouping similar quotes under broad themes while eliminating duplicates and ensuring the inclusion of data from different sources. CO and EKW provided oversight of the coding quality. Based on the deductive approach, the data analysis was guided by the study objective of exploring the knowledge, attitudes, and experiences of PCHWs in assessing and managing suicidality. No theoretical framework was used in the development of themes. The participants’ responses were initially grouped under learning, skills, perspectives, and experiences. The reactions were also categorized according to the district, level of health facility, and cadre of the participants for comparison.

## Results

A total of 18 in-depth interviews were conducted with PHCWs to explore their knowledge, experiences, and attitudes about the assessment and management of suicidality. In-depth interviews were conducted with five medical doctors, two clinical officers, two midwives, and nine nurses. The participant’s ages ranged from 28 to 49 years, while the years in service ranged from 0 to 20 (Table 1). Four themes emerged from the discussions a) Knowledge and attitudes of primary healthcare workers in the assessment and management of suicidality, b) Experiences in the assessment and management of suicidality, c) challenges faced by primary healthcare workers while assessing and managing suicidality, and d) Recommendations for improving assessment and management of suicidality in PHC.

### Theme I: Knowledge and attitudes in assessment and management of suicidality

From five questions asked, several sub-themes emerged: definition of suicide, common risk factors for suicidality, and attitudes towards suicidality.

#### Sub-theme 1: Common causes/risk factors of suicidality

On the possible causes of suicide, the participants attributed suicide to different reasons, including pre-existing mental illness, medical, social, and psychological issues, substance abuse, and genetic predisposition.

*Common causes of suicide are stress, due to many factors…. like financial issues, someone has lost a job, or somebody has made a loss, maybe in business….in married couples, you find that the man is stressing the woman. It has existed for a long time… Usually, women try to talk to the other family members, the in-laws. And when it cannot be handled at that level, they go to the law, they can go to the police, report to the LCs [local council], but if she is not helped, she may end her life…for men, the reasons usually maybe like a man gets a spouse or hears that a spouse is involved in adultery.* ***(AL010)***

Pre-existing mental illness and suicide; the participants said that a patient could come to the health facility with a pre-existing mental illness that makes them susceptible to suicide if not diagnosed and treated.

*Anything that can lead someone to go into a state of depression, then that person can think of removing [killing] themselves…. the most common cause I know as an individual is a depression.* ***(AL012)***

*…. we have seen these long-term mental disorders like bipolar disorder… Those are the most common who end up committing suicide* ***(AL018)***

Medical and social issues; the possible causes of suicidality included the unexpected realization of certain medical/health conditions, such as those who use the HIV self-test kits and find themselves HIV positive, being diagnosed with chronic illnesses such as cancer, lost jobs, or loved ones and thinking that there is no hope of continuing to live.

*.. some patients develop certain health conditions. For example, someone is diagnosed with a chronic illness, especially HIV and cancers, and if they have not gotten counseling, especially those who do self-testing for HIV, feel bad at that moment and end up committing suicide. …some have lost jobs or loved ones, especially the older adults who can no longer remarry.* ***At*** *that moment, they feel they can no longer cope and end up committing suicide (****AL004)***

Psychosocial issues and suicide: Participants also identified psychosocial issues as possible causes of suicide, especially family misunderstandings, broken relationships, and financial hardships.

*The causes are majorly some family-related issues, some social issues, and majorly quarrels within the household. It is majorly husband- wife issues and land problems….* ***(AL011)***

*… for children, it can be a separation of parents or losing a parent, and they can become frustrated due to changes at home….* ***(AL003)***

Familial risk of suicide: Some participants pointed out that suicide could be inherited

*…the other cause may be genetic … in the family, people have been committing suicide....so when you take a history, you may find that someone lost a relative in the same line….* ***(AL014)***

Substance abuse and suicide risk: The participants also identified substance abuse (alcohol or marijuana) as a possible cause of suicide.

*Most young ones take substances like Marijuana and others and resort to drinking alcohol in case of any problem. Mainly the ones we get are in that line: marriage issues, neglect, and substance abuse* ***(AL001)***

*People involved in alcohol and illicit drug use already have problems that they are failing to cope with…. so under the influence of drugs, they get the courage to commit suicide.* ***(AL004)***

#### Sub-theme 2: Attitudes of the PHCWs towards suicidality

We assessed the attitudes of the PHCWs towards those who commit or attempt suicide. Furthermore, we asked them what they, as PHCWs, would do if they had suicidal tendencies. The participants stated that the people who commit or attempt suicide are ordinary people who need help and guidance to lead everyday lives.

*I think they are usually normal people. Only that they have unresolved emotional pain because, one case of a health worker we were working with, but his wife was over-pressurizing him. He was outgoing and mixing with many ladies but the wife felt he shouldn’t move out. So, stories were that the wife resorted to witchcraft and the man could not erect when he went out with other women. He, therefore, felt that the only way to punish his wife was by taking his life. So, he decided to mix some medicines since he was a health worker and injected himself, then died. So, he was a normal person, but I think he failed to share out, and he felt there was no solution to his problem, and then he took his life. He probably would have had a better outcome if he had shared his problem with others.* ***(AL002)***

*Suicide is a psychiatric emergency. You find that they have had that thought, and if unattended, they end up committing suicide and killing themselves.* ***(AL009)***

PHCWs were asked about their personal experiences and what they would do if they had suicidal tendencies. Some of them admitted that they had had those tendencies before but sought help from their friends. Some said they did not know what to do in case they had suicidal tendencies.*..*

*It has happened to me. You know some things can get you to commit suicide. You get problems; you get loans; you get everything. Then you hear something telling you why can’t you commit suicide so that you can get relieved. I wanted to commit suicide. I had many problems, and something could come and say, “why can’t you commit suicide.” So, at night it would say, “do this so you can commit suicide.” I could talk to my friends and tell them there is something that comes and say, “why can’t you commit suicide.” You know they counsel you. And later get better and catch up (****AL016)***

*I do not know sincerely. Even the most complex challenge I have ever had. I didn’t even think about that. But you never know it can come in. But if it arrived, I would confide in someone. You know, as a person, there are people you confide in…. (****AL006)***

*In most cases, when I am depressed, I keep sharing with my friends and colleagues, like” these days I do not feel myself,” something is disturbing me. Like, you open up. It helps to open up to people who can help and talk to you. Sometimes you find people who have gone through the same experience …* ***(AL014)***

Others said they would seek counseling from their friends or professional medical personnel.

*If I developed those features, I would approach any health care provider and disclose my problem; maybe through counseling, I could be helped. Disclosure is one thing I would do* ***(AL006)***

*When I develop suicidal ideas, I share them with colleagues. Or I go to another facility, in most cases a psychiatric unit for management. At that point, most health workers go wrong by treating themselves* ***(AL005)***

### Theme II: Experiences in assessment and management of suicidality

When asked about their experiences in assessing and managing suicidality, the participants mentioned different options such as providing counseling, community outreaches, managing depression, and establishing community and health facility facilities for managing patients with suicidal tendencies.

*… those who have features of depression… we counsel them. And we manage those who have attempted suicide just like we manage patients with poisoning. And then after we counsel them and sometimes even rebuke them. … If a child has a parent, we still call the parent and talk to them. …* ***(AL003)***

Other PHCWs indicated medical treatment for the management of people with suicidal tendencies

*.. Apart from the antidepressants, we usually call the people that person is talking to or relatives and speak to them. We encourage visiting the facility for either medication or counseling. … So far, it has worked for two or three people who had poisoned themselves.…we explore and manage the root cause because people have different reasons and should be managed individually. If you identify that a person has depression, you control the depression. If it’s another psychiatric condition, you manage that cause. But specifically, you manage the root cause. If it’s a social stressor, you manage it…. AL001)*

However, the participants reported that it was hard for them to identify and get people with suicidal tendencies to open up. Overall, they agreed that all patients diagnosed with suicidality are given counselling but considered ‘difficult’ patients to manage.

*Opening up is always the problem. The moment you achieve the talking point, they burst into tears and tend to get more help. So, people who open up about their suicidality get more service compared to those who just keep quiet and have a flat affect when you are talking to them* ***(AL004****)*

*We got a case of someone who had tested (HIV) positive and had a lot of anxiety and thought of taking his life. It was a challenging experience to convince that person that he could live with the disease for a long time and do what he was supposed to do in his life. So, it was a challenging experience to convince that person to be initiated into treatment…* ***(AL012)***

The PHCWs reported that suicide could be prevented but also said that it was sometimes hard for them to assess individuals with suicidality, as shown in the quote below:

*I can only say that health workers have been neglected, yet we are also prone to suicide like any other person. …we have had 2 cases of our staff. One jumped into a septic tank … and worsened while serving at this facility; people thought he was an alcoholic. He changed in two years and committed suicide, and we were all here watching his progress* ***(AL017)***

### Theme III: Challenges in assessing and managing suicidality at PHC

We asked the PHCWs about the most challenging experiences in the assessment and management of suicidality, and the following were highlighted:

*My most challenging experience is that you talk to people you feel have suicidal intentions, treat them, and they improve, and then a few weeks or months after discharge, you hear that this person has committed suicide. So other than suicide being committed according to your assessment, when a person is at their lowest, they tend to commit suicide when according to you as a health worker, they are improving. So that is challenging. And then people who have had several attempts are counseled, but as long as you cannot solve the social factors around them, which are not always under your powers as a health worker, they eventually commit suicide. So, it’s so stressful. At times you feel you have not done your part because you have not got the results out of it (****AL004)***

Other challenges included resource constraints, the community not being supportive, low staffing levels, many inpatients due to suicidal behaviors, especially male patients, and knowledge gaps among the PHCWs.

*… One of the challenges we face is a scarcity of resources. Sometimes we don’t have antidepressants ….; other times; we are few qualified health workers who can manage such clients. So, when the one who is well versed with such clients is not around you find that it is a hassle…. And the community is non-supportive* ***(AL005)***

*The challenge is that we are not equipped with that knowledge. The knowledge we last got from school so many years ago they are the mercies we are moving on within managing such cases. Suicide is not prioritized; even if you go to the administration and say you have such a patient, they have no help for you. The male and female wards typically have the majority of such cases, but one point can disturb the whole hospital. Because the treatment is not always there, even when they are supplying those drugs, that is not a priority, yet cases of mental illness are very many….* ***(AL017)***

*We are not trained enough … we cannot go deeper … For instance, someone may say they wanted to kill themselves because their husband left them, and you cannot understand why because you have no time to go deeper and lack knowledge on what to ask. … The suicide conditions do not have obvious signs and symptoms* ***(AL001)***

### Theme IV:Recommendations for improving the assessment and management of suicidality in PHC in southwestern Uganda

The PHCWs made suggestions like general mental assessment during clinical history taking, sensitizing and creating awareness about suicidality, streamlining management of psychiatric cases in the PHC system, and equipping PHCWs in the PHC system with the knowledge to manage mental illnesses, counsel and work, and for complicated patients, referral.

The PHCWs further recommended how to address suicidality in the PHC setting. Several agreed that managing suicidality in the PHC setting is ignored, yet it is a real threat. The PHCWs requested for a system of managing suicidality to be set up, regularize community sensitization, train health workers and equip the PHC facilities with essential medicines to manage conditions (such as depression) associated with suicidality. The participants recommended improving service delivery for management and working with suicidality at staff, health facility, and community levels.

At the individual staff level, the staff needs to build a close relationship with the clients to get them to open up, but this can be achieved when they have been trained to handle people with suicidal tendencies.

*...now health workers have to be trained in identification because, at this time, identifying the signs and symptoms of suicide is very important. And ongoing health education for clients, so that the mental health with suicide is also incorporated in our health education talks…. Also, health workers being trained like ongoing training here within the facility may not need longer trainings we can even have a CME.* ***(AL010)***

*…. we need a lot of training as health workers. You find that a health center IV has only one psychiatric nurse. If they are lucky, they may have two- one registered and one enrolled. These other health center II’s or III’s don’t have a nurse, clinical officer, or doctor. And they are the majority of the health facilities compared to the hospitals…I feel we need trained health workers from the grass root.* ***(AL005)***

At the health facility level, the recommendations included giving clients health talks, quick diagnosis, mental assessment for all patients, conducting community outreaches on mental health, integrating mental health in other outreach programs, and building a framework for managing suicidality at the PHC level.

*I think the best that can be done at primary healthcare centers is counseling and referring them to the psychiatric department. Majorly that’s what can be done at the primary health care level. Counselling and then referring to the psychiatric setup. (Al006)*

*In primary health care, the setting is where we give a basic package. I think that would be ideal and we start giving health talks, and information and display pictures on the walls. Such that we make people aware because this (mental health) is silent in primary health care. It is ignored. It does not come out like these issues of sanitation, hygiene, and malaria. No. It is silent.* ***(AL013)***

At the community level, participants recommended using existing structures like Village Health Teams (VHTs) to sensitize and monitor people about suicidality so that people are observed for any suicidal changes and enrolled into the healthcare system early. They also recommended sensitization to decriminalize suicidality so that people feel safe to seek medical help when they have suicidal ideations.

*We also have the VHT [Village Health Team] system at community levels that feed into the health facility. They could also be taught some signs, such that if they see something going wrong in the community, they provide into the health facility early enough so that these people can be helped before time. Other than you hear someone has jumped from a building and is dead. Then later, the VHT tells you, “Aahh, we had observed a weird character.” But if we make them aware that if you see these signs, please let us know. Sensitization and creating awareness, such that they can adequately link them to the health facility...* ***(AL014)***

*…. decriminalizing it (suicide). Yes, for as long as it is considered a crime, people are stigmatized other than helping. Everyone who tries to commit suicide is stressed and needs to be enabled, but as long as it is criminalized, those who have attempted will never come up for help. Even the community looks at it as a criminal offense punishable by the law (****AL004)***

## Discussion

We aimed to explore the knowledge, attitudes, and experiences of primary care health workers in assessing and managing suicidality in structured primary healthcare services in southwestern Uganda using in-depth interviews. Four themes were generated I) Knowledge and attitudes of primary healthcare workers in the assessment and management of suicidality; II) Experiences of primary healthcare workers in the assessment and management of suicidality; III) challenges faced by primary healthcare workers while assessing and managing suicidality and IV) Recommendations for improving assessment and management of suicidality in PHC.

### Primary healthcare workers’ knowledge about suicidality

The primary health care workers had sufficient knowledge of the definition of suicide and the possible risk factors. These findings are similar to what has been reported in other studies on mental health knowledge [29, 30]. The knowledge about suicide was more than what has been described in previous studies for mental disorders generally [31].

The risk factors highlighted were pre-existing mental illness such as depression, substance use, unresolved psychosocial challenges, family issues, loss of jobs and loved ones, unemployment, being diagnosed with a chronic disease, and genetic predisposition. The highlighted risk factors are similar to those widely known [32–35]. The high level of knowledge among PHCWs could be due to the high burden of suicide in the communities where the health workers come from. It could also be due to their personal experiences.

### Attitudes and Challenges of PHCWs in assessing and managing suicidality

Generally, the PHCWs had positive attitudes towards the assessment and management of suicidality. The participants agreed that the people who commit or attempt suicide are normal people who need help and guidance to lead normal lives. This is in agreement with what has been reported by previous studies [36]. However, there were some PHCWs with negative attitudes. They thought the individuals required rebukes instead of psychosocial support and treatment [37, 38]. The negative attitudes may reflect the negative attitudes displayed by the government and cultural practices such as jailing individuals who have attempted and punishing the dead body of an individual who has died by suicide [8, 14–16]. Therefore, to improve PHCW’s attitudes, we need to change the attitudes of the whole system involved in mental health, starting with the government laws. They also shared their practices in the assessment and management of suicidality. The participants mentioned different options like counseling, decriminalizing suicide, community outreaches, managing depression, and establishing better community and facility facilities for managing patients with suicidal tendencies. Other PHCWs indicated medical treatment for the management of people with suicidal tendencies.

The PHCWs experienced several challenges in their practice. However, they admitted that it was hard to identify and get people with suicidal tendencies to open up. Overall, they agreed that all patients diagnosed with suicidality are given counseling. Still, getting them to open up is hard, and they consider them ‘difficult’ patients to manage. Other challenges included resource constraints, the community not being supportive, few staff, and knowledge gaps among the PHCWs.

Apart from the individuals they had assessed and managed for suicidality, some of the PHCWs themselves had had suicidality. They reported having sought help from their friends. Some said they did not know what to do in case they had suicidal tendencies. Those who were not aware of what they would do could be at increased risk of completed suicide. This finding of suicidality among health workers is not surprising because health professionals often have a higher suicide risk than other professionals [39].

The primary health care workers reported different challenging experiences, including knowledge and skills deficits, mental illness and suicide stigma, low staffing levels, and the lack of supportive community *resources.* The staff also noted predominantly having male patients admitted due to suicide behaviors*,* a finding similar to other studies done in Uganda [14, 20, 40].

### Recommendations for improving assessment and management of suicidality in PHC

PHCWs made suggestions like general mental assessment at clerking, sensitizing and creating awareness about suicidality, streamlining management of psychiatric cases in the PHC system, and equipping PHCWs in the PHC system with the knowledge to manage mental illnesses, counsel and work, and for complicated patients, referral. The PHCWs further made recommendations on the way forward to address suicidality in the PHC setting. Several agreed that managing suicidality in the PHC setting is ignored, yet it is a real threat. The PHCWs requested for a system of managing suicidality to be set up, regularize community sensitization, train health workers, and equip the PHC facilities with essential medicines to manage conditions associated with suicidality such as depression, anxiety, psychosis, etc. The participants recommended improving service delivery for management and working with suicidality at staff, health facility, and community levels. Despite the importance of a holistic approach in managing suicide and suicidality, participants only mentioned collaborations within the health system, i.e., referring to individuals with training in mental health and the VHTs. However, collaborating with the community, especially the religious leaders, NGOs, learning institutions, and law enforcement bodies, has previously been linked to reducing the burden of suicide [41].

### Clinical and policy implications

At the individual staff level, the staff needs to build a close relationship with the clients to get them to open up. However, this can only be achieved when they have been trained to handle people with suicidality and when suicidal attempts have been decriminalized. At the health facility level, mental health education should be made routine, such as giving sensitization talks, quick diagnostic assessments for all patients, conducting community mental health outreaches, integrating mental health in other outreach programs, and building a framework for managing suicidality at the PHC level.

There is a need to use existing structures like VHTs to sensitize and monitor people about suicidality so that people are observed for any suicidal changes and enrolled into the healthcare system early. They also recommended sensitization to decriminalize suicidality so that people feel safe to seek medical help when they have suicidal ideations.

Currently, in section 209 of the penal code, Uganda is one of the countries that still consider suicide a criminal offense [42]. Those who support the criminalization of suicide argue that: i) punishment is a deterrent, ii) criminal penalties for suicide attempts can express a society’s feelings of moral condemnation of certain behaviors, and iii) criminalization acts as an expression of the desire for retribution. No evidence suggests that criminalizing suicide has reduced the risk [8]. Evidence shows that criminalizing suicide hinders health-seeking behavior in a suicide crisis and receiving support to improve their mental health [43]. In addition, individuals may use more lethal means for suicide to fear the repercussions of a failed suicide attempt. In addition to punishment, a heavy stigma is attached to a failed suicide attempt. Although suicide is criminal in Uganda, punishment is seen in a small number of cases. Most individuals are taken to the hospital for assessment and care. There is a need for more research and strategies aimed at decriminalizing suicide in Uganda.

### Limitations of the study

The findings of this study should be interpreted while considering this limitation. The research team’s influence on formulating the research question, data collection procedures, purposive sampling, and using the principle of data saturation could have introduced potential bias to the study. Although the interviewers were trained in psychiatry, they did not reveal this to the interviewees. However, it could have led to “over-medicalization” and a lack of questions on addressing social factors and working with community organizations. The study was not interpreted based on a theoretical framework, an approach that might have led to a more succinct interpretation of the findings. We encourage future researchers to use such approaches in understanding the coded data.

## Conclusions and recommendations

Suicidality is commonly encountered by primary health care workers in southwestern Uganda, who have some knowledge of suicidality and risk factors, although they have limitations with its assessment and management. Improving the knowledge and attitudes of primary health care workers would be a big step towards ensuring equitable services. The lack of standard procedures to be followed by the health care workers in assessing and managing suicidality needs urgent intervention by policymakers.

## Data Availability

The data supporting the conclusions of this article are included within the paper and its additional files.
